# Community grid management is an important measure to contain the spread of novel coronavirus pneumonia (COVID-19)

**DOI:** 10.1017/S0950268820001739

**Published:** 2020-08-06

**Authors:** Chen Ling, Xianjie Wen

**Affiliations:** 1 Department of Anesthesiology, The Second People's Hospital of Foshan City, Foshan, China; 2 The Second School of Clinical Medicine, Southern Medical University, Guangzhou, China

**Keywords:** COVID-19

## Abstract

The outbreak of novel coronavirus pneumonia (coronavirus disease 2019 (COVID-19)), declared as a ‘global pandemic’ by the World Health Organization (WHO), is a public health emergency of international concern (PHEIC). The outbreak in multiple locations shows a trend of accelerating spread around the world. China has taken a series of powerful measures to contain the spread of the novel coronavirus. In response to the COVID-19 pandemic, in addition to actively finding effective treatment drugs and developing vaccines, it is more important to identify the source of infection at the community level as soon as possible to block the transmission path of the virus to prevent the spread of the pandemic. The implementation of grid management in the community and the adoption of precise management and control measures to reduce unnecessary personnel movement can effectively reduce the risk of pandemic spread. This paper mainly describes that the grid management mode can promote the refinement and comprehensiveness of community management. As a management system with potential to improve the governance ability of community affairs, it may be helpful to strengthen the prevention and control of the epidemic in the community.

## Introduction

Since the outbreak of novel coronavirus pneumonia (coronavirus disease 2019 (COVID-19)), the community is the most important place to prevent the spread of the epidemic[1, 2]. Community grid management is a new grass-roots management mode that divides urban communities into several responsibility grids, integrates dynamic community information into specific grids and completes community information collection and comprehensive governance in a comprehensive and rapid manner by virtue of the modern information technology platforms. In 2004, Beijing took the lead in implementing the community grid management mode, which achieved good results in community affairs processing, and was subsequently popularised and applied in other regions of China. During the epidemic prevention and control period, China strictly implements the community grid management mode and makes full use of modern information technology to promote the refined management of community epidemic prevention and control and contain the spread of the epidemic in the community through active intervention measures. In this paper, we shall discuss how community grid management can be utilised to build epidemic prevention and control systems, so as to fight COVID-19 and its associated challenges.

## Challenges faced by community-level epidemic prevention and control

The identities of community members are complex and the mobility of personnel is high, so there are many uncertainties. It is difficult to implement various prevention and control measures in the traditional and decentralised community management mode. The novel coronavirus pneumonia has the characteristics of long incubation period and diversified infectious pathways [3]. At present, studies have proved that patients infected with novel coronavirus are highly infectious in the early stage of infection [4]. Therefore, efficient and rapid identification of the source of infection in the community population and blocking the path of virus transmission are the decisive factors to prevent the spread of the epidemic.

## Implementation of community grid management

The community grid management is to divide the urban area into several community unit grids according to the scientific standards such as geographical layout, convenient management and integrity of management objects, and each community unit grid is divided into sub-grids again depending on the community-level information platform. Using the electronic information and data platform to accurately and quickly locate the community grid units is conducive to orderly distribution of prevention and control materials and timely resolution of public health crisis during epidemic prevention and control. Through the active inspection of the epidemic situation in the unit grid, the prevention and control measures can actively intervene, rapid response and efficient treatment, to greatly improve the execution efficiency in the unit grid. The anti-epidemic data information of each community grid unit is timely shared and interconnected. Neighbouring community grid units shall establish a regional integration cooperation mechanism, complement each other in anti-epidemic materials and human resources and implement joint prevention and control measures. The community-level information platform timely announces the progress of anti-epidemic measures. Collect residents' opinions through Internet application software and dedicated telephone lines, and respond to residents' concerns in a timely manner. The community grid management should establish a team of epidemic prevention and control personnel in the community grid unit. These personnel compose of volunteers who are familiar with the actual situation of the community and residents who have some experience in community management. The epidemic prevention materials of grid prevention and control personnel should be fully guaranteed and they should be trained in epidemic prevention knowledge. The task of grid prevention and control was clearly divided and carried out in an orderly manner to ensure that the work responsibilities are assigned to specific prevention and control personnel.

## Several suggestions for community grid management implementation

At the community level, the knowledge and measures of epidemic prevention and control should be publicised to obtain support and cooperation from residents, and all residents in the community should be fully mobilised to implement various measures of community grid management. It is suggested that the community grid prevention and control personnel be divided into several prevention and control groups, such as the entrance and exit control group, the material assurance group, the disinfection group, the grid inspection group etc. The staff of the entrance and exit control group are responsible for the pre-check and identity verification of the people entering and leaving the community, and the registration of the movement track of the people entering and leaving the community. All the people entering and leaving the community need to check their temperature, wear masks and strictly restrict the entry of outsiders into the community. The staff of the material assurance group are responsible for the purchase of protective clothing, masks, infrared temperature guns, disinfectant water and other anti-epidemic substances, arranging special personnel to be responsible for registration and distribution, helping community residents purchase daily requirements, coordinating material procurement channels and storage places and doing a good job in logistics support such as emergency supplies storage. The staff of the disinfection group are responsible for regular disinfection in public places and places with high risk of virus transmission (such as handrails and elevator buttons). The staff of the grid inspection group are responsible for the evacuation of people gathered in the community grid unit.

During the epidemic prevention and control period, relying on the big data information platform, information data is shared with disease control departments, medical institutions and anti-epidemic departments to improve prevention and control efficiency. Emergency patients in urgent need of medical treatment shall be transferred to medical institutions by specially assigned persons to reduce the risk of epidemic spread caused by residents' going out for medical treatment.

## Data Availability

Data are not used in this paper.
